# Proprioceptive engagement of the human cerebellum studied with 7T-fMRI

**DOI:** 10.1162/imag_a_00268

**Published:** 2024-08-14

**Authors:** Emma J.P. Brouwer, Nikos Priovoulos, Julie Hashimoto, Wietske van der Zwaag

**Affiliations:** Spinoza Centre for Neuroimaging, Amsterdam, The Netherlands; Computational Cognitive Neuroscience & Neuroimaging, Netherlands Institute for Neuroscience, Royal Netherlands Academy for Arts and Sciences (KNAW), Amsterdam, the Netherlands; Department of Biomedical Engineering & Physics, Amsterdam UMC, University of Amsterdam, Amsterdam, the Netherlands; Vrije Universiteit Amsterdam, Amsterdam, the Netherlands

**Keywords:** cerebellum, proprioception, fMRI, 7T-MRI, high-resolution, intrinsic 3D coordinate system

## Abstract

Proprioception, the process of perceiving our bodies in space, is a key aspect of self-perception. The cerebellar cortex is believed to play a critical role in proprioception. However, our understanding of the functional involvement of the cerebellum in proprioception remains limited due to the intricate, thin, and highly folded structure of the human cerebellar cortex, which is more challenging to resolve using in-vivo MRI compared to the cerebral cortex. In this study, we employed high-resolution, B_1_-shimmed, functional magnetic resonance imaging (fMRI) at 7T to investigate proprioceptive involvement of the cerebellum in humans. We used two tasks designed to differentially require proprioceptive information processing: midline-contralateral-finger-touch and simultaneous-unilateral-finger-flexing. We assessed responses to these tasks across three gradient directions inspired by the mesoscale cerebellar functional organisation, akin to laminar and columnar fMRI approaches in the cerebral cortex. Movements requiring higher proprioceptive engagement, in the midline-contralateral-finger-touch task, elicited stronger activations in both anterior and posterior lobe motor areas of the cerebellum (lobules V and VIIIa/b). We identified distinct activation patterns for the two tasks within these cerebellar motor regions, which may reflect differing functional roles of these motor areas. Midline-contralateral-finger-touch responses were found more medial than simultaneous-unilateral-finger-flexing responses in lobule V and deeper into the cerebellar fissures in lobule VIII. These findings contribute to a deeper understanding of cerebellar functional organisation, the cerebellar involvement in proprioception and may offer insights into addressing proprioceptive deficits associated with neurological conditions.

## 1 Introduction

Our ability to sense our bodies in space—referred to as proprioception (Bhanpuri et al., 2013)—is indispensable for every-day tasks such as maintaining posture, balancing on one leg, or simply reaching for a cup of coffee (Bloem et al., 2002). While we perform these tasks without much thought, the involvement of many brain regions is required. Proprioceptive input travels through the dorsal root ganglia through the posterior columns of the spinal cord and cuneate nucleus, through the thalamus to the primary and secondary motorsensory cortices and the cerebellum, informing these regions on the position and forces of muscles (Goble et al., 2012;Goossens et al., 2019;Iandolo et al., 2018;Marin Vargas et al., 2024;Smoth & Deacon, 1984). Although the cerebellum is known to be important for proprioceptive processing, its functional organisation is much less explored than that of the cerebral cortex. This knowledge gap arises at least partly from the inherent challenges posed by the cerebellar cortex’s intricate and highly folded structure, necessitating sophisticated high-resolution acquisition and analysis methods.

Proprioceptive deficits are often observed in patients with spinal cord injury (Dambreville et al., 2019;Formento et al., 2018;Qaiser et al., 2019) as well as several neurological conditions, including Diabetic neuropathy, Multiple Sclerosis, Parkinson’s Disease, and cerebellar ataxia (Bhanpuri et al., 2013;Ettinger et al., 2018;Iram et al., 2021;Jamali et al., 2017;Maschke et al., 2003). Even though proprioceptive deficits are common, the assessment of proprioceptive function is not trivial.Hillier et al. (2015)identified over 35 proprioceptive tasks designs, commonly using apparatuses to move limbs into desired positions, showing that somatosensory and motor sensations are unavoidable when measuring proprioception. Until recently, cerebellar damage was not thought to contribute to proprioceptive deficits (Maschke et al., 2003). This is likely due to proprioception being tested passively, that is, without movement. Proprioceptive judgement during active movement discrimination (but not passive proprioception) has since consistently been shown to be impaired in patients with cerebellar ataxia (Bhanpuri et al., 2013) or cerebellar infarcts (Bhanpuri et al., 2013;Synofzik et al., 2008;Weeks et al., 2017). This is in line with the hypothesised role of the cerebellum as a generator of internal predictions, for example of active movement outcomes (Popa & Ebner, 2019). It remains uncertain if or how the cerebellar involvement in active-movement proprioception differs from its role in movement execution: relatively low-resolution fMRI studies in humans have reported cerebellar responses in lobules V and VIII for proprioceptive processing tasks (Goossens et al., 2019;Iandolo et al., 2018) (i.e., in the same region where cerebellar BOLD response clusters appear during finger or hand movement execution (Boillat et al., 2020;King et al., 2019) and where somatotopic representations of digits have been found (Van der Zwaag et al., 2013;Wiestler et al., 2011)). It is currently unknown how proprioceptive function is organised within the cerebellar cortex in humans relative to the ‘standard’ motor clusters.

The organisation of and mechanism behind proprioceptive function has been explored in more detail in rodent models. The cerebellum receives proprioceptive information through the spinocerebellar tracts which neurons converge onto individual granule cells (Daroff & Aminoff, 2014). Electrophysiology studies have shown that mossy fibre potentials differ between input from proprioceptive and exteroceptive components (Ekerot & Larson, 1972). It was proposed that proprioceptive components activate mossy fibre tracts which are located deeper into the cerebellar fissures compared to exteroceptive components (Ekerot & Larson, 1972;Voogd, 2014). However, this is not the only functional organisation principle in the cerebellar cortex: across the phylogenetic tree a general organisation consisting of five transverse zones spanning several lobules has been reported (Craciun et al., 2018;Voogd et al., 2004). These zones are further subdivided by stripes (orthogonal to the zones) identified by differential expression of several proteins (notably zebrin II) and with distinct input and output targets (Craciun et al., 2018;Voogd et al., 2004). These functional zones and stripes have not yet been identified in humans, but could nevertheless be relevant for functional subdivisions considering their consistency across the phylogenetic tree. Additionally, histological evidence (Ekerot & Larson, 1972;Voogd, 2014) demonstrated that exteroceptive mossy fibre components are positioned superficial of the cerebellar fissures and proprioceptive mossy fibre components are found deeper in to the cerebellar fissures. Hence, for a task that requires more proprioceptive processing, a possible shift in activation is conjectured in the direction of the deeper cerebellar fissures. Alternatively, or additionally, if the underlying mechanism can be explained by functional differences between zebrin II stripes, activation differences are to be expected in the mediolateral direction. In the cerebral cortex, such mesoscale functional MRI response patterns are explored across the cortical depth and columnar directions (de Hollander et al., 2021;Dumoulin & Wandell, 2008;Huber et al., 2017;Yacoub et al., 2008). Differences between proprioceptive functional BOLD responses and motor functional BOLD responses can be explored across three gradients directions based on stripe, fissure depth, or zonal orientation. These gradient definitions are further explained in the methods section.

In order to define these gradients, high-resolution images are required. High-resolution images are more easily achieved at ultra-high field (7T). Unfortunately, at higher field strengths and given typical head coil geometries, cerebellar images can suffer from severely destructive B_1_-interference. Signals in the cerebellar area can largely be recovered using parallel transmission techniques (Oliveira et al., 2021;Padormo et al., 2016;Priovoulos et al., 2021). When pooling data across participants in high-resolution images, careful consideration is required and normalisation steps should be avoided to preserve spatial resolution (Huber et al., 2021).

In this study, we investigated the cerebellar BOLD response using a proprioceptive activation paradigm, B_1_-shimmed fMRI at 7T and data sampling along three gradient directions: across fissure depth, mediolaterally, and in the posterior/anterior direction, forming an intrinsic 3D coordinate system of local gradients. We compared responses to a simple movement to those associated with a movement more reliant on proprioception. The proprioception reliant task requires knowledge on where the fingers are in space, how to and how much to move them. We compared where and whether cerebellar activations are increased or displaced in the proprioceptive task compared to the simple movement task.

## 2 Methods

### 2.1 Experimental design

Nine healthy participants, aged 24–44 (five females), were scanned using a 7T-Philips MRI-scanner equipped with an 8Tx/32Rx (Nova Medical, USA) whole-head coil. In accordance with the declaration of Helsinki, all participants provided written informed consent and the study was approved by the local ethics committee. One participant was excluded from the study after self-reported noncompliance to the task.

**Fig. 1.**
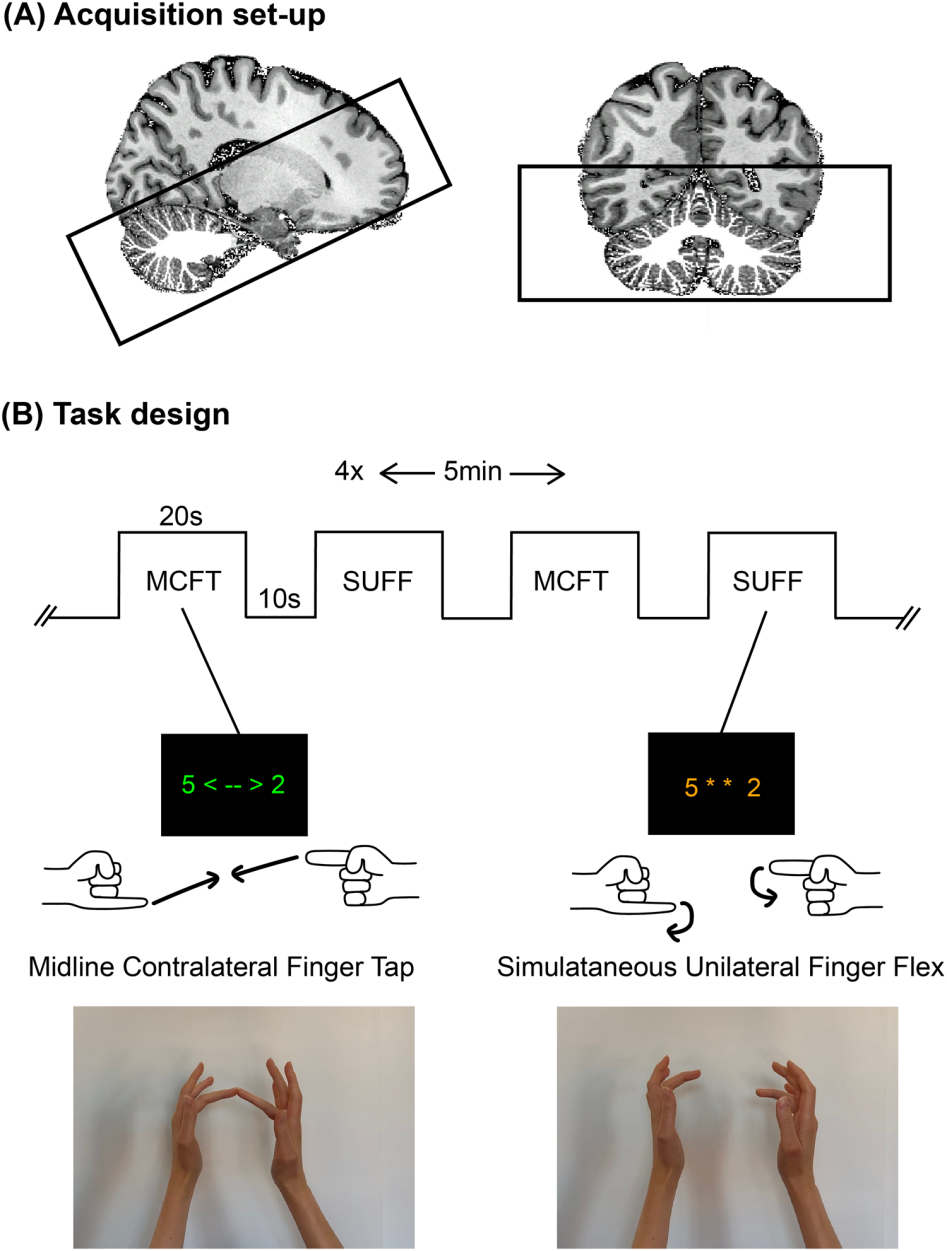
Experimental setup. (A) The whole-brain MP2RAGE (0.64mm isotropic) overlaid with the white matter cerebellar segmentation and the field of view of the functional acquisition presented with the black box. (B) The task overview of the block design including an image of the screen that participants viewed during the task. The midline contralateral finger tap (MCFT) in green and the simultaneous Unilateral Finger Flex (SUFF) in orange.

A whole-head 0.64 mm-isotropic MP2RAGE anatomical image (Marques et al., 2010) (TR/TE = 2.3 ms/6.2 ms, TI_1_/TI_2_= 800/2700, TR_volume_= 5500 ms, α = 8°/5°, FOV = 230 x 230 x 185) was acquired to generate cerebellar white matter segmentations (Fig. 1A). A DREAM B_1_^+^map (Nehrke & Börnert, 2012) was acquired, and phase modulations were calculated to optimise the B_1_^+^over the cerebellum using MRCodeTool (Tesla Dynamic Coils, Zaltbommel, NL) with the following parameters: (FOV = 224 x 224 x 168, voxel size = 3.5 mm isotropic, TR/TE = 6 ms/3 ms, α = 50°) as well as a spoiled gradient echo while transmitting with each channel separately (FOV = 224 x 224 x 168, voxel size = 3.5 mm isotropic, TR/TE = 8 ms/1.97 ms, α = 7°). A 1.0 mm-isotropic 3D-EPI slab with the following parameters: TR_volume_= 3300 ms, TR = 6.2 ms, TE = 21 ms, α = 20°, cerebellum-focussed B_1_-shim, SENSE = 2.6/3.27-AP/RL and FOV = 192 x 60 x 192 mm^3^was recorded while participants performed the task.

Each MRI session repeated functional runs of 5-minutes duration four times, alternating two tasks: (1) The Simultaneous-Unilateral-Finger-Flex (SUFF): Individual digits of each hand were moved simultaneously. (2) The Midline-Contralateral-Finger-Touch (MCFT) Individual fingers of each hand touched (Fig. 1B). For both tasks, the blocks alternated between 20 s-ON, 10 s-OFF. Pseudo-randomised finger combinations (fixed across participants) were presented for 2 s each within ON blocks (Each on block was one finger tap/touch/combination). During the MCFT blocks, the text was green and arrows indicated that participants should touch fingers of each hand to another. The fingers were placed approximately 20 cm apart, such that participants needed proprioceptive judgement to guide the respective digits towards each other. During the SUFF blocks, fingers were positioned at the same distance apart, while orange text and stars signalled untouching movements, also presented for 2 s each, again for one digit of each hand, using the same digit combinations (Fig. 1B). This ensured that participants adopted the same pace for both tasks and that both tasks were similarly challenging. As participants were placed supine in the scanner, watching the screen through a rear-viewing mirror, they were not able to see their hands, and there was no visual feedback provided during either of the two tasks. Both tasks were active motor tasks that required similar motor control. The digits and lower arms of the participants were positioned above to the torso of the participant such that no extra lower arm movement was required for MCFT compared to SUFF (a demonstration of the task can also be found in a video in theSupplementary Materials). The MCFT task requires more proprioceptive processing because the participant needs to guide the trajectory of their digits to a meeting point in the middle without any visual information. Participants practiced the task outside of the scanner to ensure familiarity with the digit numbering until performance was stabilised. After the fMRI session, participants were asked to report their compliance to the task.

### 2.2 Gradient segmentation pipeline

**Fig. 2.**
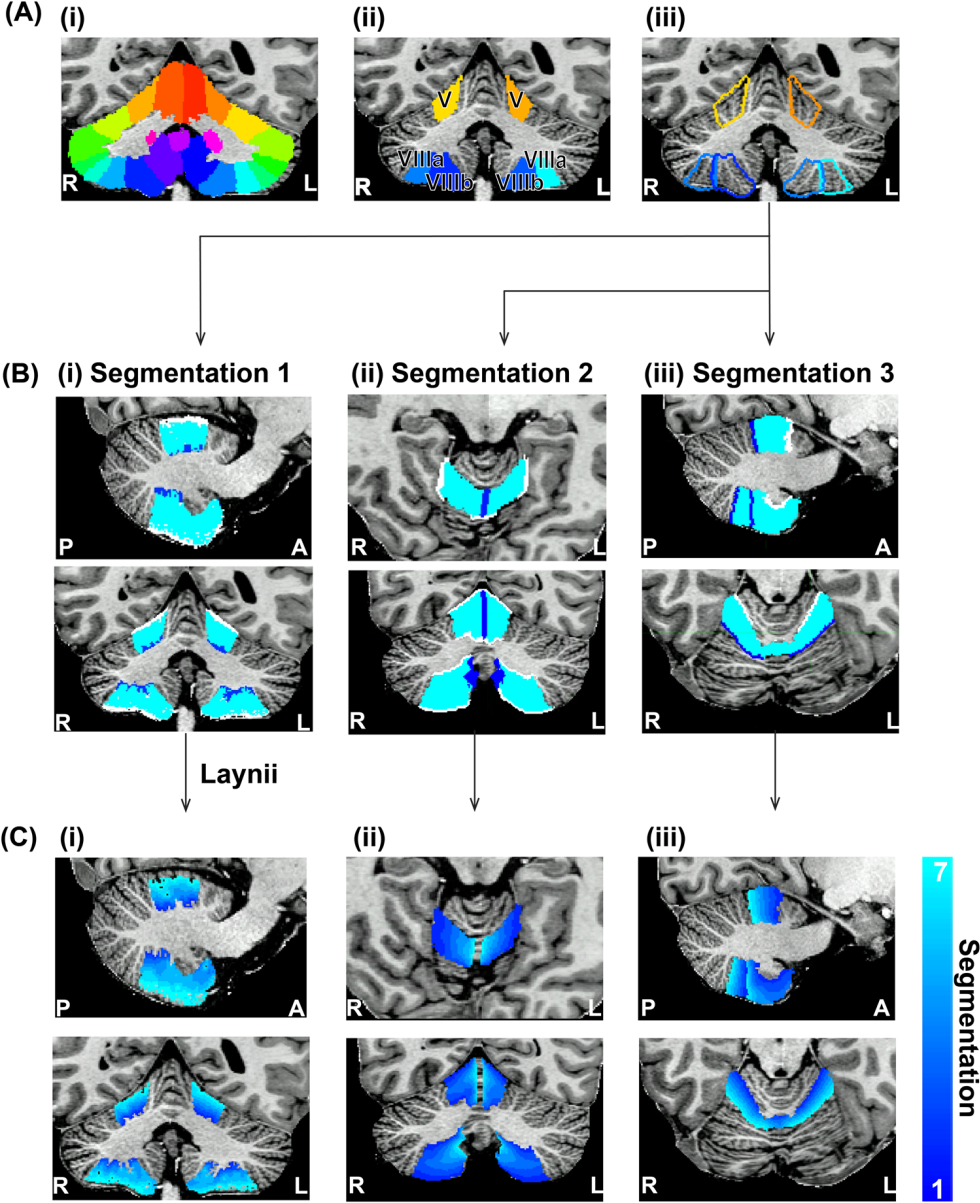
An overview of the gradient step segmentation pipeline: (A) (i) The lobular segmentation from SUIT. (ii) The extracted ROIs of lobules V, VIIIa and VIIIb (iii) The outline for the six different lobules. (B) The input to Laynii for each of the three gradients: (i) Fissure depth (ii) Mediolateral direction (iii) Posterior anterior direction. (C) The output of Laynii with seven steps presented in two orthogonal views for each of the three gradients: (i) Fissure depth (ii) Mediolateral direction (iii) Posterior anterior direction.

For each participant, lobules V, VIIIa and VIIIb (i.e., lobules wherein somatotopic representations of digits have been found and which are known to be involved in processing of proprioceptive information) were divided into seven steps along gradients in three different directions: (1) Across fissure depth (Voogd, 2014). (2) In the mediolateral direction (Craciun et al., 2018). (3) In the remaining posterior/anterior direction, all in a manner inspired by laminar imaging approaches in the cerebral cortex (Huber et al., 2021). Lobules VIIIa and VIIIb were considered separately because of their size and different folding pattern. First, the SUIT (Diedrichsen, 2006) lobule mask (Fig. 2Ai) was registered into the participant anatomical space using nonlinear registration, creating segmentations of each cerebellar lobule (Fig. 2Aii). A cerebellar white matter (WM) probability map was segmented for each participant using SPM12 (Friston, 2003) (Fig. 1A).

The deep cerebellar fissure was identified by thresholding the cerebellar WM probability map at 0.99, which was found empirically to be an appropriate threshold of the start of the white matter fissure, through visual inspection (Fig. 2Bi: dark blue). The cerebellar mask (binary mask of the entire cerebellum) was subtracted from a dilated lobule mask (the lobule mask expanded by one voxel in each direction in 3D space) to create the end point of the gradient step at the surface of the fissures (Fig. 2Bi: white).

The posterior and anterior segmentations were found using the intersection of the neighbouring lobules in the same hemisphere (Fig. 2Biii). For example, the anterior border of lobule V was identified using the overlap between lobule V and lobule IV (Fig. 2Biii: white).

The medial border was determined by the intersection of lobules in the left and right hemisphere (Fig. 2Bii: dark blue) in the participants’ space. The lateral segmentation was found by subtracting the posterior, anterior, and medial segmentations from the outlines of the lobule (coloured outlines,Fig. 2Aiii).

The segmentations in each anatomically relevant direction (Fig. 2B) were then used as input to Laynii (Huber et al., 2021) where the number of gradient steps was defined as 7. This resulted in 7 iso-distance steps for each gradient direction: over decreasing cerebellar fissure depth (Segmentation 1 = deep into the fissures, Segmentation 7 = superficial to the fissures,Fig. 2Ci), mediolateral direction (Segmentation 1 = Lateral, Segmentation 7 = Medial,Fig. 2Cii, such that the gradient steps follow the direction of the folia) and posterior anterior direction (Segmentation 1 = Posterior, Segmentation 7 = Anterior,Fig. 2Ciii).

### 2.3 fMRI analysis

The fMRI data across runs were registered in the anatomical space of each participant and were motion/distortion-corrected (MCFLIRT/TOPUP; FSL V.6.0). A first-level GLM with the following contrasts: MCFT>rest, SUFF>rest, MCFT>SUFF, (all thresholded at z > 3.1 p < 0.05) was fitted using FSL V.6.0 (Jenkinson et al., 2012). This was followed by a repeated-measures GLM for each contrast (z > 3.1 p < 0.05) to combine the four runs. The mean z-stats in the cerebellar grey matter were reported, and a paired t-test (p < 0.05) was performed between MCFT and SUFF. These, and all further additional statistical analyses, were performed using JASP (Love et al., 2019). To quantify the differences, the number of voxels containing solely MCFT activation, solely SUFF activation, or overlapping activation was reported for each participant. In addition, thresholded (z > 3.1) z-stats (MCFT>baseline and SUFF>baseline) were visually inspected in the anatomical space of each participant.

In order to assess cerebellum-wide differences in the location between the MCFT and SUFF activation, four ROIs were created in the cerebellum to examine the centre of gravity of the responses: for the right and left superior ROIs, lobules I-VIIA + Crus I were selected and for the right and left inferior ROIs, lobules VIIa and VIIIb were selected (example presented inSupplementary Fig. 1). Activation clusters (z > 0) were warped into MNI space and their centre of gravity was calculated in each mask. Paired t-tests were conducted to assess the shift in x,y,z direction.

To evaluate task activation differences along the three different gradient directions, the MCFT>SUFF contrast mean z-stats were calculated for each of the seven steps in lobules V, VIIIa and VIIb in both hemispheres. In a supplementary analysis (Supplementary Fig. 2), this was also calculated for the MCFT>REST and SUFF>REST contrast. A repeated-measures ANOVA was performed for each of the three gradient directions to compare the effect of gradient steps on mean z-stats, followed by post-hoc comparisons for significant effects between steps (p < 0.05). The Mauchly’s test was used to test the sphericity of the sample, and the Greenhouse-Geisser correction was used whenever appropriate.

## 3 Results

**Fig. 3.**
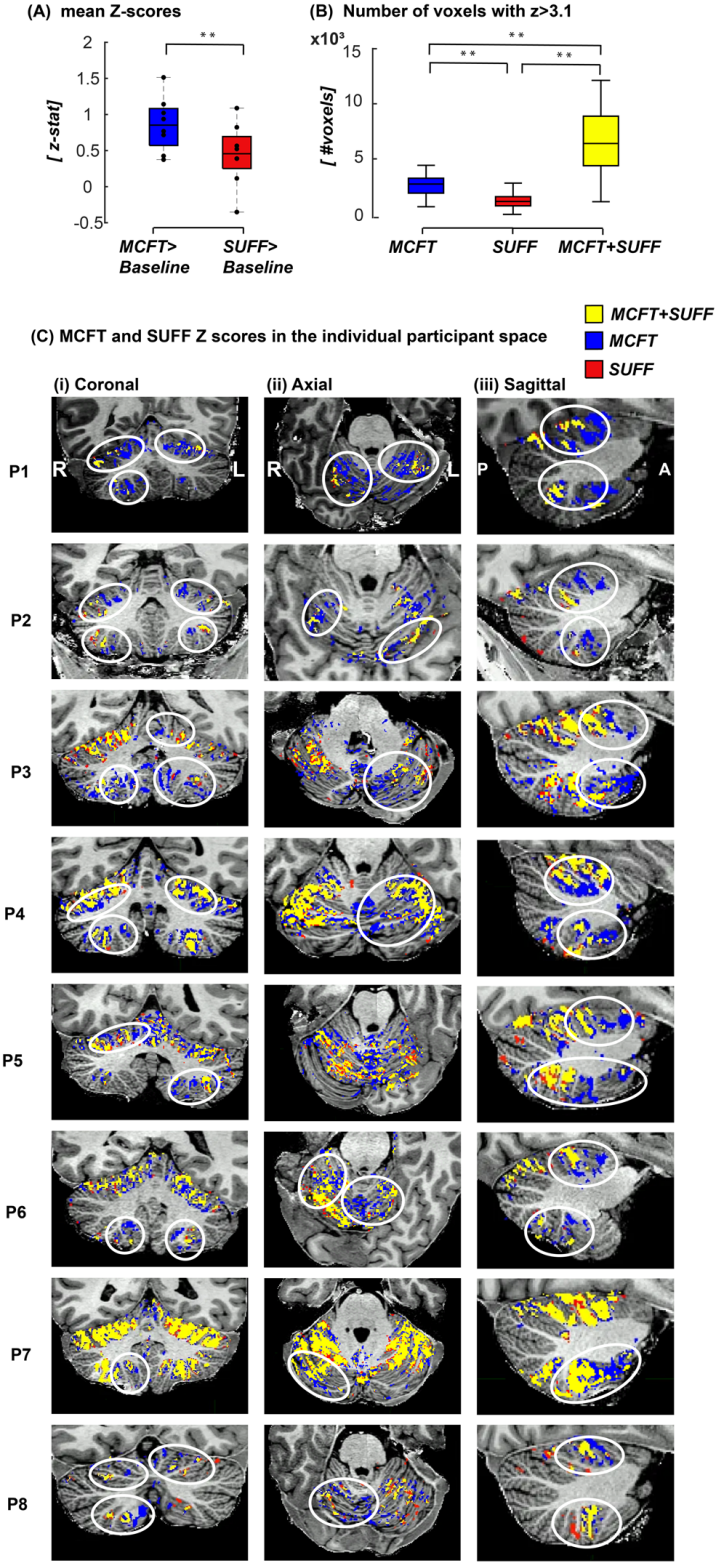
Activation maps and mean z-stats across runs (A) Mean z-scores. Note that z-stats are higher for the MCFT compared to the SUFF. (B) The number of voxels in lobules V,VIIIa/b with only MCFT activation (blue) only SUFF activation (red) overlapping MCFT and SUFF (yellow). (C) A binary representation of activation (z > 3.1) for MCFT > baseline, SUFF>baseline, and overlapping MCFT and SUFF activation presented on the (i) Corona (ii) Axial and (iii) Sagittal plane. Activation differences are highlighted with white circles. ** indicates significant difference for p < 0.01 paired t-test.

Mean z-stats were higher for the MCFT>baseline contrast compared to the SUFF>baseline contrast (Fig. 3A,Supplementary Table 1). There was a statistically significant effect of tasks on z-stats (F(1,7) = 13.927, p < 0.01). The majority of SUFF activated voxels (z > 3.1) overlapped with MCFT activation (z > 3.1), whereas MCFT activation was often present in regions where no SUFF activation was present (Fig. 3B,Supplementary Table 1). Data from each participant showed consistent activation resulting from both MCFT and SUFF tasks (Fig. 3B). Upon visual inspection in the coronal, sagittal, and axial planes of each participant, differences in activation patterns were observed: MCFT activation was found deeper into the cerebellar fissures compared to SUFF in both lobules V and VIIIa/b (Fig. 3Ci/iii). MCFT activation was located more anterior compared to SUFF activation in these slices. In lobule V, MCFT was also found more medial compared to SUFF activation (Fig. 3Cii).

**Fig. 4.**
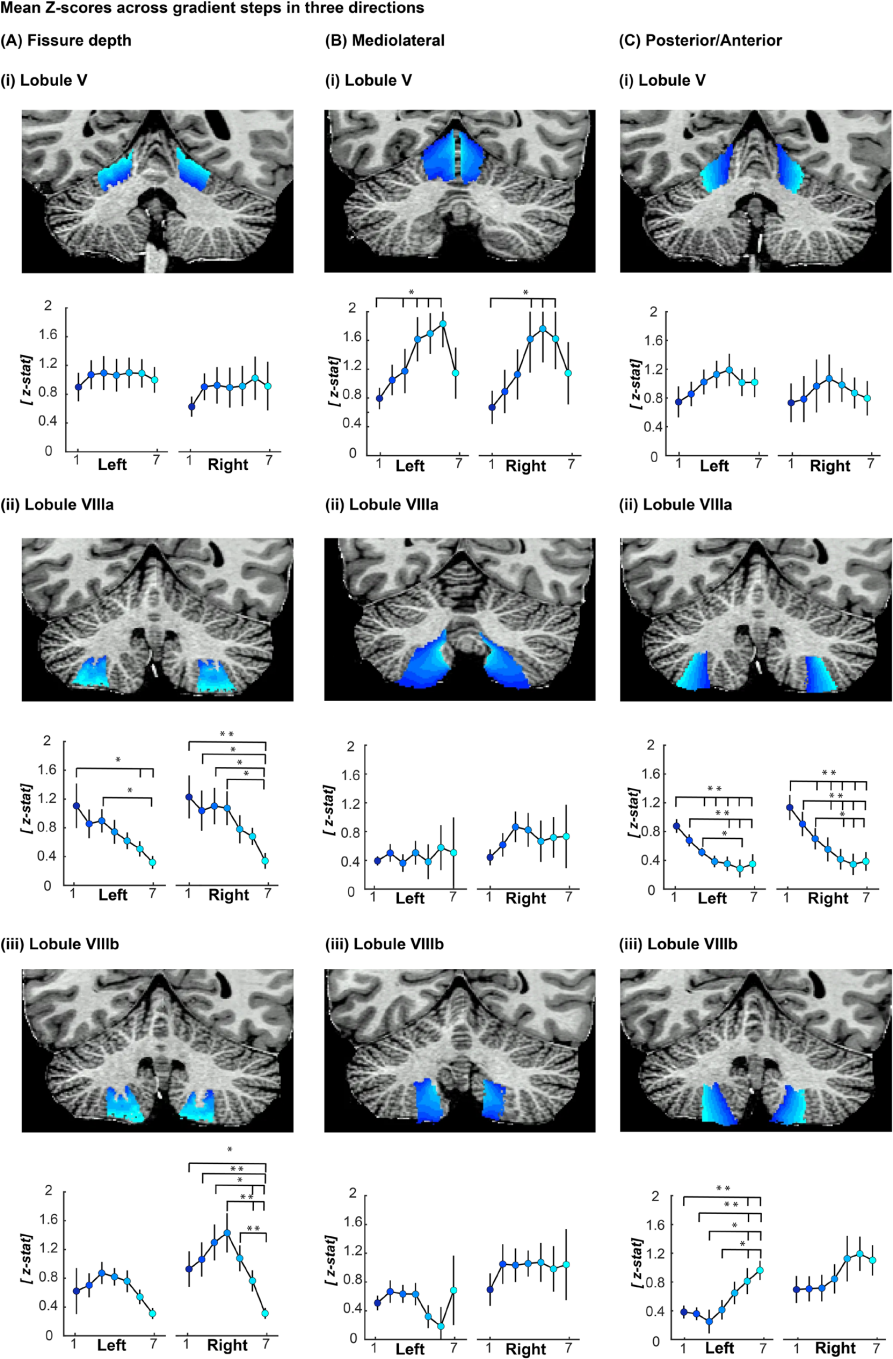
Mean z-stats across gradient steps for the MCFT > SUFF contrast. (A) z-stats across fissure depth presented for (i) Lobule V (ii) Lobule VIIIa and (iii) Lobule VIIIb. Note that in lobule VIII z-stats are higher deeper into the cerebellar fissures. (B) Z-stats across mediolateral gradient steps presented for (i) Lobule V (ii) Lobule VIIIa and (iii) Lobule VIIIb. Note that in lobule V mean z-stats are higher in the medial gradient steps. (C) z-stats across posterior/anterior gradient steps presented for (i) Lobule V (ii) Lobule VIIIa and (iii) Lobule VIIIb. Note that in Lobule VIIIa mean z-stats are higher in the anterior gradient steps and in lobule VIIIb mean z-stas are higher in the posterior gradient steps. * Indicates a significant difference for p < 0.05 paired t-test, ** indicates a significant difference for p < 0.01 t-test.

A paired t-test revealed a significant difference between the MCFT and SUFF activation COG location (z > 0) in the x direction in all four cerebellar quadrants (Table 1,Supplementary Table 3). In line with the visual inspection, significant z-stat differences were found across segmentations, all indicated with * inFigure 4: In lobule VIIIa/b, a significant effect of gradient steps on MCFT>SUFF z-stats was found with higher z-scores deeper into the cerebellar fissures compared to the superficial gradient steps (Fig. 4A; lobule VIIIa right (F(1.77,12.4) = 4.260, p < 0.043), lobule VIIIa left (F(1.56,10.94) = 4.274,p < 0.05), lobule VIIIb right (F(2.39,16.74) = 9.427,p < 0.001)).

**Table 1.** COG shift in the x,y,z direction in MNI space.

ROI	Mean shift x (mm)	σ	p	Mean shift y (mm)	σ	p	Mean shift z (mm)	σ	p
RU	1.15	1.32	0.04*	0.75	0.70	0.02*	0.79	1.04	0.07
RL	0.85	0.79	0.02*	0.91	0.97	0.03*	0.34	0.85	0.29
LU	-1.96	1.18	<0.001**	0.50	1.47	0.37	0.93	0.94	0.03*
LL	-0.69	0.80	0.04*	0.37	0.92	0.29	0.05	0.68	0.84

Significant p-values are presented in bold, * indicates a significant difference for p < 0.05 paired t-test, ** indicates a significant difference for p < 0.01 t-test.

In lobule V, a significant effect of gradient steps on MCFT>SUFF z-scores was found with higher z-stats more medial compared to the lateral gradient steps (Fig. 4B; lobule V right (F(3.02,21.12) = 5.97, p < 0.004), lobule V left (F(1.737,12.16) = 4.851, p < 0.032)).

Anterior-posterior gradient step differences were inconsistent across lobules and found in opposite directions. In lobule V, no significant differences were found between gradient steps in the posterior anterior gradient. Namely, in lobule VIIIa, a significant effect of gradient steps on MCFT>SUFF z-stats was found with higher z-stats more anterior compared to the posterior gradient steps (Fig. 4C; lobule VIIIa left (F(2.38,16.65) = 18.32, p < 0.001), lobule VIIIa right (F(2.30,16.10) = 22.10,p < 0.001)). Lobule VIIIb showed an opposite gradient: a significant effect of gradient steps on MCFT>SUFF z-stats was found with higher z-stats more posterior compared to the anterior gradient steps (Fig. 4C; VIIIb left (F(2.14,15.01) = 10.30, p < 0.001)). Post-hoc Holm t-tests between steps are presented inFigure 4andSupplementary Table 2for all three directions.

## 4 Discussion

We employed individually B_1_-shimmed 7T fMRI to map the evolution of BOLD activation along three different, anatomically relevant, gradient directions within lobules V and VIII during a proprioceptive task. Our subject specific processing allowed us to avoid the detrimental smoothing effect of a group normalisation step and increase our sensitivity to subtle differences between the active motor movements in SUFF and the more proprioceptively-engaging MCFT tasks. This method revealed consistent differences in the responses in lobules V and VIIIa/b along the three gradient directions.

Our gradient selection was inspired by the neocortical functional organisation patterns. In-vivo neuroscientific research has utilised advances in (f)MRI resolution to move past gross sulcal function descriptions to explore the mesoscale layer- and column-specific functional organisation of the neocortex (de Hollander et al., 2021;Dumoulin, 2017;Huber et al., 2017;Yacoub et al., 2008). The cerebellum is known to have its own functional organisation principles, in the form of stripes, zones, and possibly fissure-depth (Craciun et al., 2018;Giovannucci et al., 2017;Voogd, 2014). Here, we utilised a local gradient-approach to explore BOLD responses along relevant directions in the human cerebellum. While we utilised lobular boundaries for the gradient definitions in our study, it is essential to acknowledge that these boundaries do not necessarily align with functional boundaries (Buckner et al., 2011;King et al., 2019). In our present analysis, this is evident inFigure 3, where responses extend beyond the confines of lobules V and VIII. However, we validated the shift we observed through a Center of Gravity (CoG) analysis conducted in larger regions of interest (ROIs) including lobules I-VIII and Crus (seeTable 1).

In accordance with our hypothesis, responses to a more proprioceptive task were found more medial compared to responses to a motor-only task. An analogy for this mediolateral shift can be found in the cerebellar language areas, where more schematic integrations occur in more lateral areas and more concrete, motor-relevant tasks such as vowel generation engage more medial areas (D’Mello et al., 2020). This mediolateral organisation could also reflect the para-sagittally oriented stripes with distinct function that are consistently observed in animals across the phylogeny (Ekerot & Larson, 1972;Voogd, 2014). Note that this mediolateral shift was not observed in the posterior lobe motor area in lobule VIII, but only in the motor area of lobule V. This aligns well with what is observed in primates: for example, in marmosets the stripe-like functional organisation is much less prominent in lobule VIII compared to lobule V, potentially due to the increased anatomical complexity of lobule VIII (D’Mello et al., 2020).

Our results revealed a specific organisation of proprioceptive activation across fissure depth, with deeper areas of the posterior cerebellar lobe seemingly more engaged with processing of proprioceptive information compared to the more superficial areas engaged in a simple motor task. These results are in agreement with earlier studies (Voogd, 2014) that found proprioceptive cerebellar mossy fibre systems to terminate deeper into the cerebellar fissures in rodents.

Moreover, we found a change in activation strength across the anterior-posterior direction across participants in lobule VIIIa, where activation with a proprioceptive component was found more anterior compared to activation less reliant on proprioception. Lobule VIIIb showed the opposite; proprioceptive activation was found more posterior. This might simply be the reflection of the shift in the fissure depth direction, as these steps are not completely perpendicular.

Our COG results revealed a consistent shift in the x direction in all four quadrants. This shift roughly corresponds to the mediolateral gradient where we also detected a shift in lobule V. The z direction of the COG roughly corresponds to the fissure depth gradient, and the y direction is similar to the posterior anterior gradient. However, the apparent difference we find in the COG in the z and y directions cannot be explicitly transformed to the gradient results due to the non-cartesian, non-perpendicular nature of the subject specific gradients.

While our approach allowed the assessment of cerebellar activation across different gradient directions, limitations do exist. In this study, we assessed our functional results within the context of cerebellar anatomical structures. The gradients used here may not reflect the exact functional structures of the cerebellum but they do indicate that there are differences along these axes for specific task features. Additionally, higher-resolution acquisitions (close to 0.2 mm isotropic) have been shown to be necessary to fully disentangle the anatomical structures of the cerebellum (Sereno et al., 2020). This could allow segmentations fully faithful to the anatomy and therefore gradient definitions with higher specificity. Such resolutions are currently out of reach for in-vivo human imaging though recent advances are closing this gap (Priovoulos et al., 2023). Third, this study employed a relatively small sample size, focused on within-sample reproducibility. This was necessitated by the relatively complex acquisition protocol and individually-specific processing. Increased sample size can improve generalisability of the obtained results to the general human population and guide follow-up clinical studies in diseases.

Fourth, the literature remains divided on tasks for measuring proprioceptive function (Hillier et al., 2015). Specifically, since the cerebellum is involved with active proprioception (Bhanpuri et al., 2013;Synofzik et al., 2008;Weeks et al., 2017), there is an inherent challenge of disentangling proprioceptive function from motor execution, since these are unavoidable during active proprioceptive tasks. Here, we kept the position and movement as similar as possible between the two tasks, thereby controlling for motor movement between the two tasks. This is similar to earlier matching tasks performed with the foot (Iandolo et al., 2018) and arm (Dukelow et al., 2010). However, a slight difference in tactile sensation between these tasks remains: the MCFT includes finger touching, whereas the SUFF does not. We chose this approach to ensure participants received feedback about their movements, maintaining consistency across the task for all participants. While this means we cannot entirely rule out the possibility that the observed shift is partly due to this tactile stimulation difference, we believe this is highly unlikely given earlier research presented byWiestler et al. (2011), where sensory and motor clusters in the cerebellum were shown to be highly overlapping (Wiestler et al., 2011). Therefore, we interpret the measured shift between SUFF and MCFT responses as due to the increased demand for proprioceptive information during the MCFT task and not due to a difference in tactile input.

Although our study has demonstrated differences in cerebellar responses, our imaging slab did not cover the cerebral cortex. While previous studies have investigated the relation between the cerebellum and the cerebral cortex across different cognitive domain (King et al., 2023;Nakai & Nishimoto, 2022;Ren et al., 2019), to this date, no fMRI studies using a proprioceptive paradigm and covering both the cortex and the cerebellum have been performed. Investigating the entire circuit as a whole would be a relevant next step. Traditionally, closed-loop interactions between distinct cerebellar and cerebrocortical sites have been assumed to exist, though recent fMRI (King et al., 2019) evidence shows that convergent inputs from multiple cortical regions can better predict cerebellar functions (King et al., 2023), which potentially expands for proprioception as well. A theta burst stimulation study (Mirdamadi & Block, 2021) revealed that the cerebellum and S1 are involved with different aspects of proprioceptive function during skill leaning, further supporting this idea. Further exploration into the underlying organisational principles governing these networks requires true whole-brain coverage as well as high spatial resolution.

Overall, our experiments confirm the cerebellum is an important target for the assessment of proprioceptive deficits. The differences between active motor movement and proprioceptive function are subtle and fMRI methods can help to identify these with high spatial specificity: particularly in combination with 7T imaging methods (Priovoulos et al., 2023), we can potentially further clarify how variations in proprioceptive processing within the cerebellum may contribute to conditions such as multiple sclerosis, cerebellar ataxia, and Parkinson’s disease and their relation to disruptions in cerebellar proprioceptive and motor pathways (Goossens et al., 2019;Jamali et al., 2017;Konczak et al., 2010;Schmahmann, 2004). Better understanding of these relationships may provide insights into the pathophysiology of these disorders and create opportunities for the development of targeted therapeutic strategies.

## 5 Conclusion

Our methods allowed us to inspect 7T BOLD responses to two tasks which differed in the amount of proprioceptive demand in motor areas of the human cerebellum, across three gradient directions based on the individual’s cerebellar anatomy. This subject-specific, local gradient approach allowed investigation of cerebellar function responses in a biologically relevant coordinate system similar to laminar fMRI in the neocortex. Movements with higher proprioceptive engagement yielded stronger activations in the cerebellum than a simple motor task, and these were found more medial in lobule V and deeper into the cerebellar fissures in lobule VIII, in agreement with cerebellar mesoscale organisation patterns.

## Supplementary Material

Supplementary Material

MCFT

SUFF

## Data Availability

The data that support the findings of this study are available from the corresponding author, upon reasonable request.
