# Supplementary material for: Proprioceptive engagement of the human cerebellum studied with 7T-fMRI
Source: Imaging Neurosci (Camb). 2024 Aug 14;2:imag-2-00268. doi: 10.1162/imag_a_00268 (PMC12290657; doi:10.1162/imag_a_00268)
Supplement: Supplementary Material [file imag_a_00268-supp.pdf]

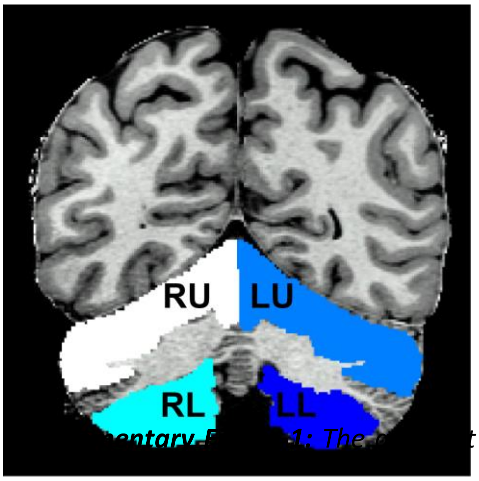

[illegible]entary-F[illegible]1: The [illegible]t steps used for the CoG calculations in the cerebellum. The upper gradient steps (right upper (RU) and left upper (LU) include lobules I-VIIA + CrusI and the lower gradient steps (right lower (LU) and left lower (LL)) include lobules VII + VIIIA/B.

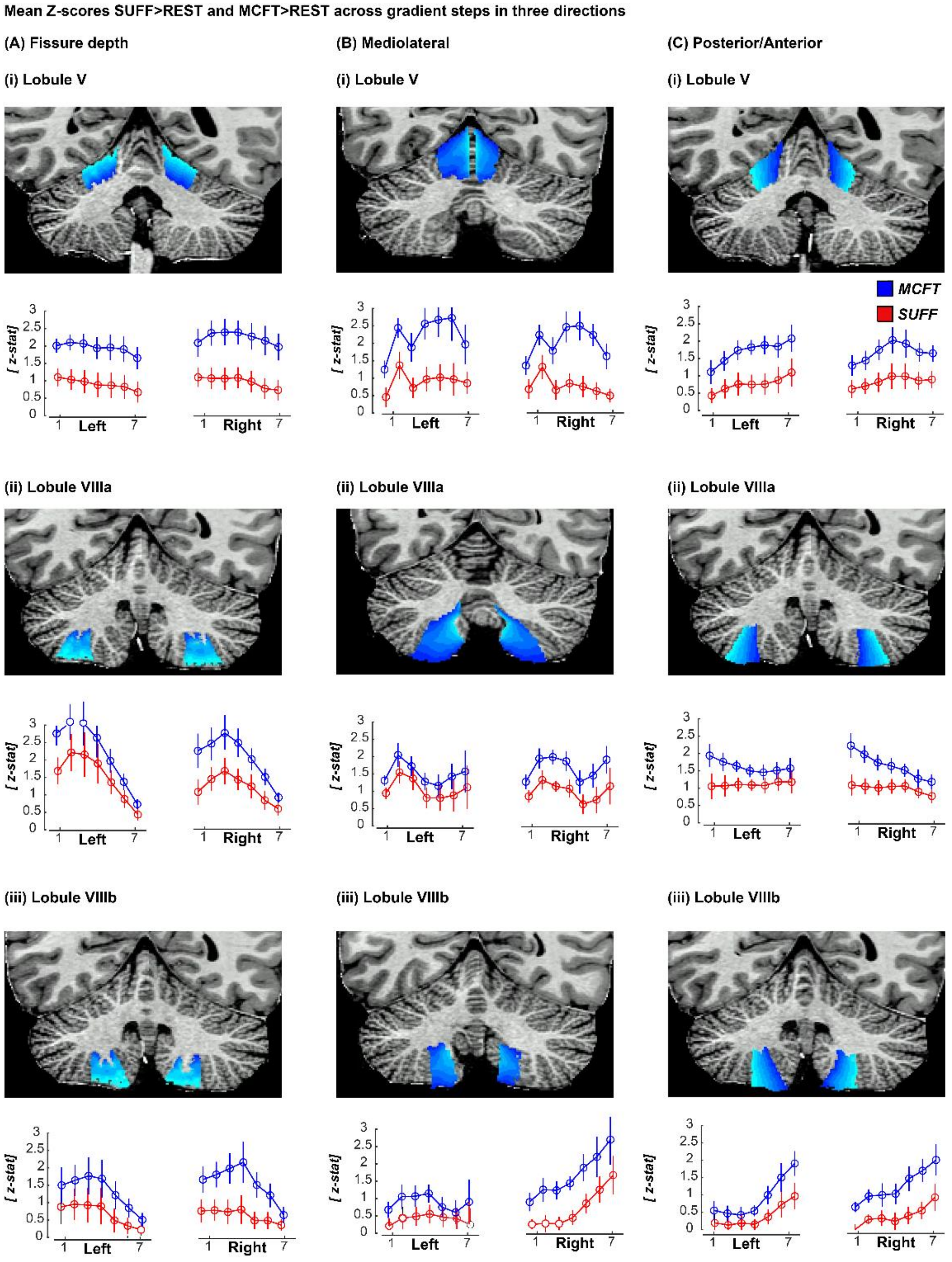

__Supplementary Figure 2:__ Mean z-stats across gradient steps for the MCFT > REST (Blue) and SUFF>REST (Red) contrast. (A) Z-stats across fissure depth (B) Z-stats across mediolateral gradient steps. (C) Z-stats across posterior/anterior gradient steps.

Supplementary Table 1: Statistical tests performed for figure 3A/B

Table 1.1: Mean z-stats repeated measures Anova

Task		Mean Difference	SE	t	p_{holm}
MCFT	SUFF	0.177	0.047	3.732	0.007**

Table 1.2: Number of voxels (z>3.1) repeated measures Anova

Task		Mean Difference	SE	t	p_{holm}
MCFT	SUFF	1456.292	367.235	3.966	< .001**
	MCFT + SUFF	-3793.313	367.235	-10.329	< .001**
SUFF	MCFT + SUFF	-5249.604	367.235	-14.295	< .001**
Note. P-value adjusted for comparing a family of 3					

Supplementary Table 2: Mean z-stats across gradient steps repeated measures Anova

Table 2.1 Fissure depth

Lobule V Left: Within Subjects Effects						
Cases	Sphericity Correction	Sum of Squares	df	Mean Square	F	p
Gradient step	Greenhouse-Geisser	0.252	2.294	0.110	0.645	0.558
Residuals	Greenhouse-Geisser	2.730	16.059	0.170		
Note. Type III Sum of Squares						

Lobule V right: Within Subjects Effects						
Cases	Sphericity Correction	Sum of Squares	df	Mean Square	F	p
Gradient step	Greenhouse-Geisser	0.714	2.390	0.299	1.273	0.311
Residuals	Greenhouse-Geisser	3.927	16.728	0.235		
Note. Type III Sum of Squares						

Lobule VIIIa left : Within Subjects Effects						
Cases	Sphericity Correction	Sum of Squares	df	Mean Square	F	p
Gradient step	Greenhouse-Geisser	3.325	1.563	2.127	4.274	0.050*
Residuals	Greenhouse-Geisser	5.445	10.940	0.498		
Note. Type III Sum of Squares						

Lobule VIIIa left: Post Hoc Comparisons					
Gradient step		Mean Difference	SE	t	p_{holm}
1	2	0.251	0.180	1.392	1.000
	3	0.209	0.180	1.160	1.000
	4	0.362	0.180	2.009	0.713
	5	0.487	0.180	2.705	0.167
	6	0.600	0.180	3.331	0.036*
	7	0.787	0.180	4.369	0.002**
2	3	-0.042	0.180	-0.231	1.000
	4	0.111	0.180	0.618	1.000
	5	0.236	0.180	1.313	1.000
	6	0.349	0.180	1.940	0.769
	7	0.536	0.180	2.977	0.087
3	4	0.153	0.180	0.849	1.000
	5	0.278	0.180	1.544	1.000
	6	0.391	0.180	2.171	0.535
	7	0.578	0.180	3.209	0.049*
4	5	0.125	0.180	0.695	1.000
	6	0.238	0.180	1.322	1.000
	7	0.425	0.180	2.359	0.368
5	6	0.113	0.180	0.626	1.000
	7	0.300	0.180	1.664	1.000
6	7	0.187	0.180	1.038	1.000
Note. P-value adjusted for comparing a family of 21					

Lobule VIIIa Right: Within Subjects Effects						
**Cases**	**Sphericity Correction**	**Sum of Squares**	**df**	**Mean Square**	**F**	**p**
Gradient step	Greenhouse-Geisser	4.589	1.772	2.589	4.260	0.043*
Residuals	Greenhouse-Geisser	7.541	12.406	0.608		
Note. Type III Sum of Squares						

Lobule VIIIa Right: Post Hoc Comparisons					
**Gradient step**		**Mean Difference**	**SE**	**t**	**p_{holm}**
1	2	0.190	0.212	0.897	1.000
	3	0.126	0.212	0.594	1.000
	4	0.156	0.212	0.737	1.000
	5	0.449	0.212	2.117	0.643
	6	0.548	0.212	2.585	0.226
	7	0.889	0.212	4.194	0.003**
2	3	-0.064	0.212	-0.304	1.000
	4	-0.034	0.212	-0.160	1.000
	5	0.258	0.212	1.220	1.000
	6	0.358	0.212	1.687	1.000
	7	0.699	0.212	3.297	0.036*
3	4	0.030	0.212	0.144	1.000
	5	0.323	0.212	1.524	1.000
	6	0.422	0.212	1.991	0.742
	7	0.763	0.212	3.601	0.017*
4	5	0.292	0.212	1.380	1.000
	6	0.391	0.212	1.848	0.932
	7	0.732	0.212	3.457	0.024*
5	6	0.099	0.212	0.468	1.000
	7	0.440	0.212	2.077	0.659
6	7	0.341	0.212	1.610	1.000
Note. P-value adjusted for comparing a family of 21					

Lobule VIIIb Left : Within Subjects Effects						
**Cases**	**Sphericity Correction**	**Sum of Squares**	**df**	**Mean Square**	**F**	**p**
Gradient step	Greenhouse-Geisser	1.765	1.349	1.308	3.063	0.106
Residuals	Greenhouse-Geisser	4.033	9.445	0.427		
Note. Type III Sum of Squares						

Lobule VIIIb Right : Within Subjects Effects							
**Cases**	**Sphericity Correction**	**Sum of Squares**	**df**	**Mean Square**	**F**	**p**	**ω^2**
Gradient step	Greenhouse-Geisser	6.525	2.392	2.728	9.428	0.001**	0.225
Residuals	Greenhouse-Geisser	4.844	16.742	0.289			
Note. Type III Sum of Squares							

Lobule VIIIb Right : Post Hoc Comparisons					
**Gradient step**		**Mean Difference**	**SE**	**t**	**p_{holm}**
1	2	-0.133	0.170	-0.782	1.000
	3	-0.370	0.170	-2.182	0.417
	4	-0.502	0.170	-2.957	0.071
	5	-0.150	0.170	-0.885	1.000
	6	0.164	0.170	0.967	1.000
	7	0.616	0.170	3.627	0.012*
2	3	-0.238	0.170	-1.399	1.000
	4	-0.369	0.170	-2.174	0.417
	5	-0.017	0.170	-0.102	1.000
	6	0.297	0.170	1.750	0.700
	7	0.749	0.170	4.409	0.001*
3	4	-0.132	0.170	-0.775	1.000

	5	0.220	0.170	1.297	1.000
	6	0.535	0.170	3.149	0.045*
	7	0.986	0.170	5.808	< .001**
4	5	0.352	0.170	2.072	0.444
	6	0.666	0.170	3.924	0.005*
	7	1.118	0.170	6.583	< .001**
5	6	0.314	0.170	1.852	0.640
	7	0.766	0.170	4.511	< .001**
6	7	0.452	0.170	2.659	0.143
Note. P-value adjusted for comparing a family of 21					

Table 2.2 Mediolateral:

**Lobule V left: Within Subjects Effects**						
**Cases**	**Sphericity Correction**	**Sum of Squares**	**df**	**Mean Square**	**F**	**p**
Gradient step	Greenhouse-Geisser	7.144	1.737	4.112	4.851	0.032*
Residuals	Greenhouse-Geisser	10.309	12.161	0.848		
Note. Type III Sum of Squares						

**Lobule V left: Post Hoc Comparisons**					
**Gradient Step**		**Mean Difference**	**SE**	**t**	**p_{holm}**
1	2	-0.256	0.248	-1.033	1.000
	3	-0.381	0.248	-1.540	1.000
	4	-0.823	0.248	-3.324	0.035*
	5	-0.903	0.248	-3.645	0.015*
	6	-1.039	0.248	-4.193	0.003*
	7	-0.353	0.248	-1.424	1.000
2	3	-0.126	0.248	-0.507	1.000
	4	-0.568	0.248	-2.291	0.379
	5	-0.647	0.248	-2.612	0.186
	6	-0.783	0.248	-3.160	0.053
	7	-0.097	0.248	-0.392	1.000
3	4	-0.442	0.248	-1.784	0.817
	5	-0.521	0.248	-2.105	0.496
	6	-0.657	0.248	-2.653	0.180
	7	0.029	0.248	0.116	1.000
4	5	-0.080	0.248	-0.321	1.000
	6	-0.215	0.248	-0.869	1.000
	7	0.471	0.248	1.899	0.708
5	6	-0.136	0.248	-0.548	1.000
	7	0.550	0.248	2.221	0.414
6	7	0.686	0.248	2.768	0.142
Note. P-value adjusted for comparing a family of 21					

**Lobule V Right: Within Subjects Effects**						
**Cases**	**Sphericity Correction**	**Sum of Squares**	**df**	**Mean Square**	**F**	**p**
Gradient step	Greenhouse-Geisser	8.241	3.018	2.731	5.966	0.004**
Residuals	Greenhouse-Geisser	9.669	21.124	0.458		
Note. Type III Sum of Squares						

**Lobule V Right: Post Hoc Comparisons**					
**Gradient Step**		**Mean Difference**	**SE**	**t**	**p_{holm}**
1	2	-0.221	0.240	-0.920	1.000
	3	-0.458	0.240	-1.910	0.587
	4	-0.951	0.240	-3.963	0.005**
	5	-1.093	0.240	-4.555	< .001**
	6	-0.954	0.240	-3.975	0.005**

	7	-0.475	0.240	-1.982	0.587
2	3	-0.238	0.240	-0.991	1.000
	4	-0.730	0.240	-3.044	0.066
	5	-0.872	0.240	-3.635	0.014*
	6	-0.733	0.240	-3.055	0.066
	7	-0.255	0.240	-1.062	1.000
3	4	-0.493	0.240	-2.053	0.587
	5	-0.634	0.240	-2.645	0.172
	6	-0.495	0.240	-2.065	0.587
	7	-0.017	0.240	-0.072	1.000
4	5	-0.142	0.240	-0.592	1.000
	6	-0.003	0.240	-0.012	1.000
	7	0.475	0.240	1.981	0.587
5	6	0.139	0.240	0.580	1.000
	7	0.617	0.240	2.573	0.192
6	7	0.478	0.240	1.993	0.587
Note. P-value adjusted for comparing a family of 21					

**Lobule VIIIa left : Within Subjects Effects**						
**Cases**	**Sphericity Correction**	**Sum of Squares**	**df**	**Mean Square**	**F**	**p**
Gradient step	Greenhouse-Geisser	0.321	1.355	0.237	0.150	0.781
Residuals	Greenhouse-Geisser	15.007	9.488	1.582		
Note. Type III Sum of Squares						

**Lobule VIIIa Right : Within Subjects Effects**						
**Cases**	**Sphericity Correction**	**Sum of Squares**	**df**	**Mean Square**	**F**	**p**
Gradient step	Greenhouse-Geisser	0.947	1.845	0.513	0.894	0.425
Residuals	Greenhouse-Geisser	7.411	12.918	0.574		
Note. Type III Sum of Squares						

**Lobule VIIIb Left: Within Subjects Effects**						
**Cases**	**Sphericity Correction**	**Sum of Squares**	**df**	**Mean Square**	**F**	**p**
Gradient step	Greenhouse-Geisser	1.817	1.631	1.114	1.198	0.327
Residuals	Greenhouse-Geisser	10.622	11.414	0.931		
Note. Type III Sum of Squares						

**Lobule VIIIb Right: Within Subjects Effects**						
**Cases**	**Sphericity Correction**	**Sum of Squares**	**df**	**Mean Square**	**F**	**p**
Gradient step	Greenhouse-Geisser	0.858	1.389	0.618	0.447	0.583
Residuals	Greenhouse-Geisser	13.448	9.721	1.383		

Table 2.3 Posterior Anterior:

**Lobule V Left: Within Subjects Effects**						
**Cases**	**Sphericity Correction**	**Sum of Squares**	**df**	**Mean Square**	**F**	**p**
Gradient step	Greenhouse-Geisser	1.113	2.389	0.466	3.011	0.069
Residuals	Greenhouse-Geisser	2.588	16.725	0.155		
Note. Type III Sum of Squares						

**Lobule V Right: Within Subjects Effects**						
**Cases**	**Sphericity Correction**	**Sum of Squares**	**df**	**Mean Square**	**F**	**p**
Gradient step	Greenhouse-Geisser	0.730	2.354	0.310	1.532	0.245
Residuals	Greenhouse-Geisser	3.337	16.480	0.202		
Note. Type III Sum of Squares						

Lobule VIIIa Left: Within Subjects Effects						
**Cases**	**Sphericity Correction**	**Sum of Squares**	**df**	**Mean Square**	**F**	**p**
Gradient step	Greenhouse-Geisser	2.216	2.378	0.932	18.319	< .001
Residuals	Greenhouse-Geisser	0.847	16.648	0.051		
Note. Type III Sum of Squares						

Lobule VIIIa Left: Post Hoc Comparisons					
**Gradient Step**		**Mean Difference**	**SE**	**t**	**p_{holm}**
1	2	0.200	0.071	2.817	0.081
	3	0.364	0.071	5.127	< .001
	4	0.493	0.071	6.951	< .001
	5	0.524	0.071	7.384	< .001
	6	0.591	0.071	8.329	< .001
	7	0.528	0.071	7.434	< .001
2	3	0.164	0.071	2.309	0.259
	4	0.293	0.071	4.134	0.002
	5	0.324	0.071	4.567	< .001
	6	0.391	0.071	5.512	< .001
	7	0.328	0.071	4.616	< .001
3	4	0.130	0.071	1.824	0.526
	5	0.160	0.071	2.258	0.259
	6	0.227	0.071	3.203	0.031
	7	0.164	0.071	2.307	0.259
4	5	0.031	0.071	0.433	1.000
	6	0.098	0.071	1.378	1.000
	7	0.034	0.071	0.482	1.000
5	6	0.067	0.071	0.945	1.000
	7	0.003	0.071	0.049	1.000
6	7	-0.064	0.071	-0.896	1.000
Note. P-value adjusted for comparing a family of 21					

Lobule VIIIa Right: Within Subjects Effects						
**Cases**	**Sphericity Correction**	**Sum of Squares**	**df**	**Mean Square**	**F**	**p**
Gradient step	Greenhouse-Geisser	4.235	2.300	1.842	22.091	< .001
Residuals	Greenhouse-Geisser	1.342	16.097	0.083		
Note. Type III Sum of Squares						

Lobule VIIIa Right: Post Hoc Comparisons					
**Gradient Step**		**Mean Difference**	**SE**	**t**	**p_{holm}**
1	2	0.231	0.089	2.583	0.120
	3	0.435	0.089	4.869	< .001
	4	0.583	0.089	6.522	< .001
	5	0.720	0.089	8.061	< .001
	6	0.791	0.089	8.850	< .001
	7	0.750	0.089	8.395	< .001
2	3	0.204	0.089	2.286	0.198
	4	0.352	0.089	3.939	0.004
	5	0.490	0.089	5.478	< .001
	6	0.560	0.089	6.267	< .001
	7	0.519	0.089	5.812	< .001
3	4	0.148	0.089	1.653	0.529
	5	0.285	0.089	3.192	0.027
	6	0.356	0.089	3.981	0.003
	7	0.315	0.089	3.526	0.011
4	5	0.138	0.089	1.539	0.529
	6	0.208	0.089	2.328	0.198
	7	0.167	0.089	1.873	0.408

5	6	0.070	0.089	0.789	1.000
	7	0.030	0.089	0.334	1.000
6	7	-0.041	0.089	-0.455	1.000

Note. P-value adjusted for comparing a family of 21

Lobule VIIIb left: Within Subjects Effects

Cases	Sphericity Correction	Sum of Squares	df	Mean Square	F	p
Gradient step	Greenhouse-Geisser	3.366	2.144	1.570	10.295	0.001**
Residuals	Greenhouse-Geisser	2.288	15.010	0.152		

Note. Type III Sum of Squares

Lobule VIIIb left: Post Hoc Comparisons

Gradient step		Mean Difference	SE	t	p_{holm}
1	2	0.023	0.117	0.197	1.000
	3	0.132	0.117	1.130	1.000
	4	-0.032	0.117	-0.270	1.000
	5	-0.266	0.117	-2.281	0.277
	6	-0.429	0.117	-3.679	0.010
	7	-0.580	0.117	-4.968	< .001
2	3	0.109	0.117	0.934	1.000
	4	-0.054	0.117	-0.466	1.000
	5	-0.289	0.117	-2.477	0.191
	6	-0.452	0.117	-3.875	0.006
	7	-0.603	0.117	-5.164	< .001
3	4	-0.163	0.117	-1.400	1.000
	5	-0.398	0.117	-3.411	0.020
	6	-0.561	0.117	-4.809	< .001
	7	-0.712	0.117	-6.098	< .001
4	5	-0.235	0.117	-2.011	0.457
	6	-0.398	0.117	-3.409	0.020
	7	-0.548	0.117	-4.698	< .001
5	6	-0.163	0.117	-1.398	1.000
	7	-0.314	0.117	-2.687	0.123
6	7	-0.150	0.117	-1.289	1.000

Note. P-value adjusted for comparing a family of 21

Lobule VIIIb right: Within Subjects Effects

Cases	Sphericity Correction	Sum of Squares	df	Mean Square	F	p
Gradient step	Greenhouse-Geisser	2.343	2.794	0.838	3.537	0.036*
Residuals	Greenhouse-Geisser	4.636	19.560	0.237		

Note. Type III Sum of Squares

Lobule VIIIb right: Post Hoc Comparisons

Gradient step		Mean Difference	SE	t	p_{holm}
1	2	-0.010	0.166	-0.063	1.000
	3	-0.020	0.166	-0.119	1.000
	4	-0.148	0.166	-0.888	1.000
	5	-0.430	0.166	-2.590	0.236
	6	-0.497	0.166	-2.990	0.098
	7	-0.406	0.166	-2.444	0.282
2	3	-0.009	0.166	-0.056	1.000
	4	-0.137	0.166	-0.825	1.000
	5	-0.420	0.166	-2.527	0.261
	6	-0.486	0.166	-2.927	0.110
	7	-0.396	0.166	-2.381	0.306
3	4	-0.128	0.166	-0.769	1.000
	5	-0.410	0.166	-2.471	0.282

	6	-0.477	0.166	-2.870	0.121
	7	-0.386	0.166	-2.325	0.325
4	5	-0.283	0.166	-1.702	1.000
	6	-0.349	0.166	-2.101	0.500
	7	-0.258	0.166	-1.555	1.000
5	6	-0.066	0.166	-0.400	1.000
	7	0.024	0.166	0.146	1.000
6	7	0.091	0.166	0.546	1.000
Note. P-value adjusted for comparing a family of 21					

Supplementary Table 3: COG coordinates in MNI space of the MCFT>REST and SUFF>REST Z-scores (Z>0)

	**MCFT**			**SUFF**					
**ROI**	**x**	**y**	**z**	**x**	**y**	**z**	**xdif**	**ydiff**	**zdiff**
**Right Upper**									
s1	67.52	67.55	50.30	63.87	65.96	47.65	3.66	1.59	2.65
s2	63.01	67.20	47.61	61.82	67.25	46.09	1.19	-0.05	1.52
s3	64.15	67.34	47.70	63.67	66.53	47.68	0.47	0.80	0.02
s4	63.25	67.25	47.80	62.46	66.27	48.04	0.78	0.98	-0.24
s5	65.98	67.84	49.20	66.62	66.95	48.82	-0.64	0.89	0.38
s6	63.31	69.34	47.99	62.50	67.68	47.72	0.81	1.66	0.27
s7	63.31	69.15	48.25	62.83	69.42	48.24	0.48	-0.27	0.00
s8	63.82	66.03	48.15	61.40	65.67	46.45	2.42	0.36	1.70
**Right Lower**									
s1	67.49	66.27	21.30	66.19	64.85	21.10	1.30	1.43	0.20
s2	67.54	65.99	22.03	65.89	65.85	21.29	1.65	0.14	0.74
s3	66.52	68.45	21.50	65.73	66.54	21.73	0.78	1.90	-0.24
s4	66.65	66.43	22.21	65.60	66.57	20.87	1.05	-0.14	1.34
s5	69.58	64.97	22.31	70.26	62.91	21.81	-0.69	2.06	0.50
s6	68.47	67.97	20.85	67.77	66.55	19.56	0.70	1.43	1.28
s7	69.06	66.79	21.83	68.80	65.80	21.64	0.26	0.99	0.18
s8	68.34	66.28	21.28	66.59	66.82	22.54	1.75	-0.54	-1.26
**Left Lower**									
s1	114.96	68.37	49.51	119.02	69.94	47.39	-4.06	-1.57	2.12
s2	117.03	66.85	48.09	119.84	67.25	45.95	-2.80	-0.39	2.14
s3	114.64	67.03	48.09	116.38	67.22	47.41	-1.75	-0.20	0.68
s4	116.85	68.53	48.44	117.84	68.76	48.57	-0.99	-0.23	-0.13
s5	115.53	68.33	48.90	117.46	65.59	47.68	-1.93	2.74	1.22
s6	116.85	68.88	47.40	118.22	66.88	47.16	-1.37	1.99	0.25
s7	117.27	69.38	47.85	117.51	69.42	48.08	-0.24	-0.04	-0.23
s8	114.87	67.68	48.73	117.41	65.99	47.34	-2.54	1.69	1.39
**Left Lower**									
s1	113.19	70.08	22.85	114.72	70.10	22.17	-1.54	-0.02	0.68
s2	113.94	68.62	22.24	114.80	68.31	21.90	-0.86	0.32	0.34
s3	112.91	67.84	21.25	112.81	66.30	21.05	0.11	1.53	0.20
s4	114.53	68.16	21.38	115.78	69.26	20.87	-1.25	-1.10	0.51
s5	114.02	68.30	22.69	113.64	66.56	23.38	0.38	1.74	-0.69
s6	112.68	66.57	21.15	114.48	66.83	20.56	-1.80	-0.26	0.59
s7	113.30	68.78	22.33	113.35	68.37	22.30	-0.05	0.41	0.03
s8	113.23	68.34	21.04	113.76	67.97	22.28	-0.54	0.37	-1.25
